# Bridging Gaps in Tuberculosis Control: A Culturally Competent Approach for Tribal Populations in India

**DOI:** 10.7759/cureus.80689

**Published:** 2025-03-16

**Authors:** Akash G Prabhune, Priyanka Dadha

**Affiliations:** 1 Health and Information Technology, ADMIRE Centre for Advancing Digital Health, Institute of Health Management Research-Bangalore (IIHMR-B), Bengaluru, IND; 2 Nutrition, Institute for Public Health and Medicine, Northwestern University Feinberg School of Medicine, Chicago, USA

**Keywords:** culturally competent healthcare, national tuberculosis elimination program (ntep), socio-cultural barriers to healthcar, tribal populations in india, tuberculosis (tb) elimination

## Abstract

Tuberculosis (TB) remains a major health challenge in India, with a significant burden among tribal populations. These communities experience disproportionately high TB prevalence due to factors such as geographical isolation, socio-economic challenges, and cultural practices, further worsened by malnutrition, overcrowded living conditions, and limited awareness. Despite efforts under national programs aimed at eliminating TB, various socio-cultural and logistical barriers continue to impede progress in tribal regions.

This discussion underscores the need for culturally sensitive healthcare approaches to effectively address these challenges. A structured framework focusing on cultural awareness, knowledge, sensitivity, and competence is recommended to develop tailored interventions. Key strategies include training healthcare workers to understand tribal customs, creating accessible educational materials, involving tribal leaders in stigma-reduction efforts, and integrating traditional practices with modern healthcare systems.

To achieve impactful outcomes, it is essential to enhance healthcare infrastructure, implement customized awareness campaigns, address underlying socio-economic issues, and leverage technology for better outreach and adherence. Real-world examples from tribal regions illustrate both advancements and ongoing gaps in TB care. A collaborative effort among various stakeholders is crucial to bridge healthcare disparities and empower tribal communities in the fight against TB, ultimately contributing to the national goal of its elimination while improving the overall well-being of these populations.

## Editorial

Tuberculosis (TB) remains a global health crisis, with India accounting for 27% of the world’s 10 million TB cases and 25% of deaths. Among India's diverse population, tribal communities, known as Adivasis, face disproportionately high TB prevalence (703 cases per 100,000), though data remain sparse [1]. Tribal communities encounter unique healthcare challenges stemming from geographical remoteness, socio-economic disparities, cultural practices, and a lack of infrastructure. Factors such as poverty, malnutrition, overcrowded living conditions, and limited awareness exacerbate TB prevalence [2]. Addressing these requires culturally sensitive and equitable solutions that integrate traditional practices and bolster tribal resilience.

The National Tuberculosis Elimination Program (NTEP), rebranded from the Revised National TB Control Programme (RNTCP), targets TB elimination by 2025 through strategies such as Directly Observed Treatment, Short-Course (DOTS), active case-finding, and drug-resistant TB management [3]. The Indian Council of Medical Research (ICMR) and Central TB Division (CTD) have been instrumental in addressing TB among tribal populations through tailored outreach programs, awareness campaigns, and mobile clinics. These efforts aim to reduce stigma, enhance early diagnosis, and improve healthcare access [4]. While progress has been made, gaps persist in overcoming socio-cultural and logistical barriers in tribal regions.

Geographical isolation, limited transport options, and poorly equipped health systems hinder timely TB diagnosis and treatment in tribal areas. Many rely on private practitioners [5] or traditional healers [6], delaying care. Financial barriers and inadequate awareness about TB symptoms exacerbate these challenges [7]. Cultural norms often limit women's healthcare-seeking behaviors, while men may seek care more readily [8,9]. Traditional remedies, beliefs in spiritual causes, and caregiving responsibilities among women delay professional medical intervention. High migration rates disrupt treatment adherence, and misconceptions about TB symptoms often delay diagnosis [10,11], Substance abuse, prevalent in some tribal communities, complicates recovery [12]. Tailored awareness campaigns and educational initiatives are essential to address these gaps [9,13].

Isolated regions like Odisha [5, 14] and Madhya Pradesh [9] lack sufficient healthcare infrastructure. Poor transportation and infrequent visits by health workers contribute to delayed diagnoses and incomplete treatments [5,9]. Poverty and malnutrition weaken immune defenses, while overcrowded and unsanitary living conditions increase TB transmission risks. Tribal populations often lack access to proper sanitation and safe drinking water, worsening the disease burden [13]. The stigma around TB, compounded by traditional beliefs [7] and substance abuse [9], prevents many from seeking care. Faith healers and misconceptions about spiritual causes delay effective treatment [7]. Table 1 highlights the key differences between the tribal and non-tribal populations of India, as derived from the literature.

**Table 1:** Comparative analysis of tuberculosis burden in tribal and non-tribal populations in India Sources: [1-7]

Category	Tribal populations	Non-tribal populations
Tuberculosis (TB) prevalence rate	703 per 100,000	311 per 100,000
Multidrug-resistant TB (MDR-TB) cases	Higher due to incomplete treatments	Lower due to better healthcare adherence
Healthcare access	Limited health infrastructure, reliance on traditional healers	Better access to healthcare facilities
Malnutrition as a risk factor	High prevalence leading to weakened immunity	Comparatively lower impact
Stigma and awareness	Higher stigma and misconceptions	Better awareness and early detection

Culturally competent healthcare ensures patient-centered, respectful care tailored to tribal populations' unique values. Integrating cultural sensitivity into TB programs improves communication, reduces stigma, and enhances treatment adherence. Existing gaps in addressing TB among tribal populations highlight critical challenges. Language barriers persist due to the lack of linguistically adapted materials and interpreters, hindering the effectiveness of awareness campaigns. Additionally, a cultural mismatch exists, as many tribal groups prioritize traditional remedies, while TB programs often fail to incorporate culturally tailored approaches. Gender disparities further exacerbate the issue, with women facing restricted mobility and caregiving responsibilities that limit their access to healthcare services.

Case studies illustrate these challenges. Among the Saharia Tribe in Madhya Pradesh, factors such as migration, poverty, and reliance on traditional healers continue to hinder effective TB care. While mobile clinics and health workers have improved outreach, these efforts require further scaling to meet the community's needs [7,10,11]. Similarly, in the Odisha tribal belt, the reliance on private practitioners and traditional healers, compounded by gender roles, delays timely medical intervention. Tribal-friendly health camps have shown promise in addressing these barriers, demonstrating the potential for culturally sensitive healthcare initiatives to enhance TB care in these regions [5,14]. Additionally, state-wise TB burden varies significantly, with Odisha reporting the highest prevalence among tribal communities (803 per 100,000), while states like Jammu and Kashmir have lower prevalence rates (127 per 100,000) [15].

Evidence suggests that TB-related deaths are significantly higher in tribal areas due to delayed healthcare access and inadequate treatment coverage. A study in India found that tribal populations residing in remote regions exhibit TB mortality rates considerably higher than the national average due to diagnostic delays, treatment discontinuation, and reliance on traditional healers [16]. Similar trends are observed globally, with Indigenous communities in Canada and the United States experiencing TB mortality rates several times higher than their non-Indigenous counterparts due to geographical isolation and underfunded healthcare services [17]. These findings underscore the urgent need for culturally competent healthcare models that integrate localized outreach, community engagement, and tailored TB interventions.

While previous studies have highlighted the high TB burden among tribal populations in India, much of the existing literature focuses on epidemiological trends and general programmatic interventions without fully addressing the socio-cultural dimensions that influence healthcare access and treatment adherence. This paper adds to the discourse by providing a structured framework based on cultural awareness, knowledge, sensitivity, and competence, which has been largely underexplored in TB control efforts within tribal settings. Unlike prior studies that primarily emphasize biomedical and infrastructural challenges, this paper integrates culturally competent strategies, drawing on real-world case studies and global best practices, to propose context-specific, community-driven interventions. By doing so, it not only identifies key gaps in the NTEP outreach to tribal communities but also presents a scalable, policy-relevant model that can enhance TB control efforts in similar marginalized populations worldwide.

Cultural competence framework

Using the Papadopoulos model [18], we propose a cultural competence framework that addresses four levels of cultural competence (Table 2).

**Table 2:** A summary of actions and justifications

Framework level	Key actions	Justification from content
Cultural awareness	Train healthcare workers on biases and involve traditional healers	Cultural mismatch and stigma prevent healthcare uptake; trust-building requires respect for traditional practices.
Cultural knowledge	Develop linguistically adapted materials; and conduct qualitative studies	Language barriers and reliance on home remedies delay care-seeking behavior.
Cultural sensitivity	Engage tribal leaders; reduce stigma; and offer flexible care models	Stigma and misconceptions about TB as spiritual punishment require culturally sensitive education programs.
Cultural competence	Train traditional healers; employ tribal health workers; and holistic care	Poverty, malnutrition, and migration disrupt adherence; community engagement improves outreach and outcomes.

To effectively address TB among tribal populations, a culturally competent approach is essential. This involves employing local tribal individuals as health workers to bridge cultural and linguistic gaps, which can be further strengthened through intersectoral coordination. Government skill councils can play a crucial role by imparting healthcare job skills to young people from tribal communities and linking them with existing National Skill Council programs. By integrating tailored skill training courses, such initiatives can enhance workforce capacity, ensuring culturally competent healthcare delivery in tribal regions. Such collaborative efforts between health and skill development sectors can significantly improve the implementation and effectiveness of TB control strategies, leading to better healthcare access, stronger community trust, and improved health outcomes. Developing cultural knowledge is equally critical, including creating linguistically and visually adapted TB education materials and researching tribal beliefs and gender-specific challenges to design tailored interventions. Enhancing cultural sensitivity requires engaging tribal leaders in stigma-reduction campaigns, offering mobile clinics with flexible schedules, and providing nutritional support to align with tribal lifestyles. Lastly, applying cultural competence entails training traditional healers to recognize TB symptoms and refer patients, employing local tribal individuals as health workers to bridge cultural and linguistic gaps, and addressing socio-economic determinants like malnutrition and poverty through integrated development programs. Together, these measures promote respectful, patient-centered care, ensuring that TB interventions are effective and equitable.

Advocating for a comprehensive, culturally tailored approach in TB programs is essential to address the unique challenges faced by diverse communities. Evidence indicates that integrating cultural and religious contexts into TB interventions enhances their effectiveness. Table 3 presents the importance of culturally tailored TB interventions, which is evident in various global case studies.

**Table 3:** Evidence of culturally tailored approaches in tuberculosis programs

Country/Region	Culturally tailored approach	Outcome	Reference
Indonesia (Mentawai and Solok regions)	Used religious leaders, informal leaders, and traditional music to raise awareness and reduce stigma.	Increased tuberculosis (TB) awareness, testing, and treatment adherence.	Machmud et al. [19]
Pakistan (Rawalpindi's transgender community)	Conducted TB screening camps using portable X-ray machines and artificial intelligence (AI)-based analysis to make healthcare more accessible for transgender individuals.	Improved TB detection and treatment adherence.	The Guardian, 2025 [20]
Zambia (Gender-specific strategies)	Gender-sensitive interventions: community-based TB testing for men and incentivized, peer-based case-finding for women.	Enhanced diagnosis rates and treatment adherence through gender-specific strategies.	Kerkhoff et al. [21]

Policy recommendations

Enhance Healthcare Infrastructure

Establish more local clinics and mobile health units in remote areas to improve access to TB diagnosis and treatment. Operation ASHA in India and Cambodia successfully deployed community-based DOTS providers in slum and tribal areas, ensuring treatment adherence through doorstep TB care [22]. Integrating traditional and modern healthcare systems, as seen in Peru’s Partners In Health model, can enhance accessibility and trust among tribal populations [23].

Culturally Tailored Awareness Campaigns

Translate TB awareness materials into local languages and engage tribal leaders to debunk myths and stigma. In Indonesia, using religious leaders and traditional healers in TB awareness campaigns significantly improved treatment adherence and reduced stigma [19]. Incorporating visual aids and culturally familiar storytelling, like in Zambia’s gender-sensitive TB outreach programs, can enhance community engagement and early diagnosis [21].

Target Socio-Economic Factors

Address underlying determinants by providing financial support, improving living conditions, and implementing substance abuse rehabilitation programs. The Navajo Nation TB Control Program in the U.S. successfully linked housing support with TB care, ensuring better adherence to long-term treatment [24]. Similarly, Peru’s TB program integrates nutrition support with TB treatment, recognizing malnutrition as a major risk factor for treatment failure [23].

Leverage Technology

Utilize telemedicine and mobile health solutions for remote monitoring, treatment adherence, and patient follow-ups. India’s Nikshay Ecosystem, a digital TB monitoring platform, has streamlined patient tracking, financial incentives, and community engagement [25].

Conclusion and the way forward

Addressing TB in tribal populations requires a multifaceted approach, integrating cultural competence, socio-economic support, and strengthening healthcare infrastructure. Collaborative efforts by ICMR, CTD, non-governmental organizations (NGOs), and tribal leaders have shown promising outcomes in improving TB diagnosis, treatment adherence, and awareness. However, sustained action is crucial to bridge healthcare gaps. By enhancing access to TB care, reducing socio-economic disparities, and implementing culturally adapted interventions, India can make substantial progress toward TB elimination in tribal regions and achieve its 2025 goal.

The insights and policy recommendations presented in this paper provide an evidence-based foundation for future research and programmatic interventions, contributing to a comprehensive understanding of TB control in tribal communities. With culturally sensitive strategies, India can make significant strides toward eliminating TB in tribal regions and achieving its 2025 goal.
